# Alternative Method for Obtaining Human-Induced Pluripotent Stem Cell Lines and Three-Dimensional Growth: A Simplified, Passage-Free Approach that Minimizes Labor

**DOI:** 10.21769/BioProtoc.5081

**Published:** 2024-10-05

**Authors:** Masaya Tsukamoto, Tomoyuki Kawasaki, Akihiro Umezawa, Hidenori Akutsu

**Affiliations:** Center for Regenerative Medicine, National Center for Child Health and Development, 2-10-1 Okura, Setagaya, Tokyo, Japan

**Keywords:** Bioreactor, Enzyme-free passaging, iPSCs, Reprogramming, 3D culture

## Abstract

Induced pluripotent stem cells (iPSCs) hold significant promise for numerous applications in regenerative medicine, disease modeling, and drug discovery. However, the conventional workflow for iPSC generation, with cells grown under two-dimensional conditions, presents several challenges, including the need for specialized scientific skills such as morphologically assessing and picking colonies and removing differentiated cells during the establishment phase. Furthermore, maintaining established iPSCs in three-dimensional culture systems, while offering scalability, necessitates an enzymatic dissociation step for their further growth in a complex and time-consuming protocol. In this study, we introduce a novel approach to address these challenges by reprogramming somatic cells grown under three-dimensional conditions as spheres using a bioreactor, thereby eliminating the need for two-dimensional culture and colony picking. The iPSCs generated in this study were maintained under three-dimensional conditions simply by transferring spheres to the next bioreactor, without the need for an enzymatic dissociation step. This streamlined method simplifies the workflow, reduces technical variability and labor, and paves the way for future advancements in iPSC research and its wider applications.

Key features

• Establishment of induced pluripotent stem cells in a three-dimensional environment.

• Maintenance and cryopreservation of iPSCs without the need for a dissociation step.

## Graphical overview


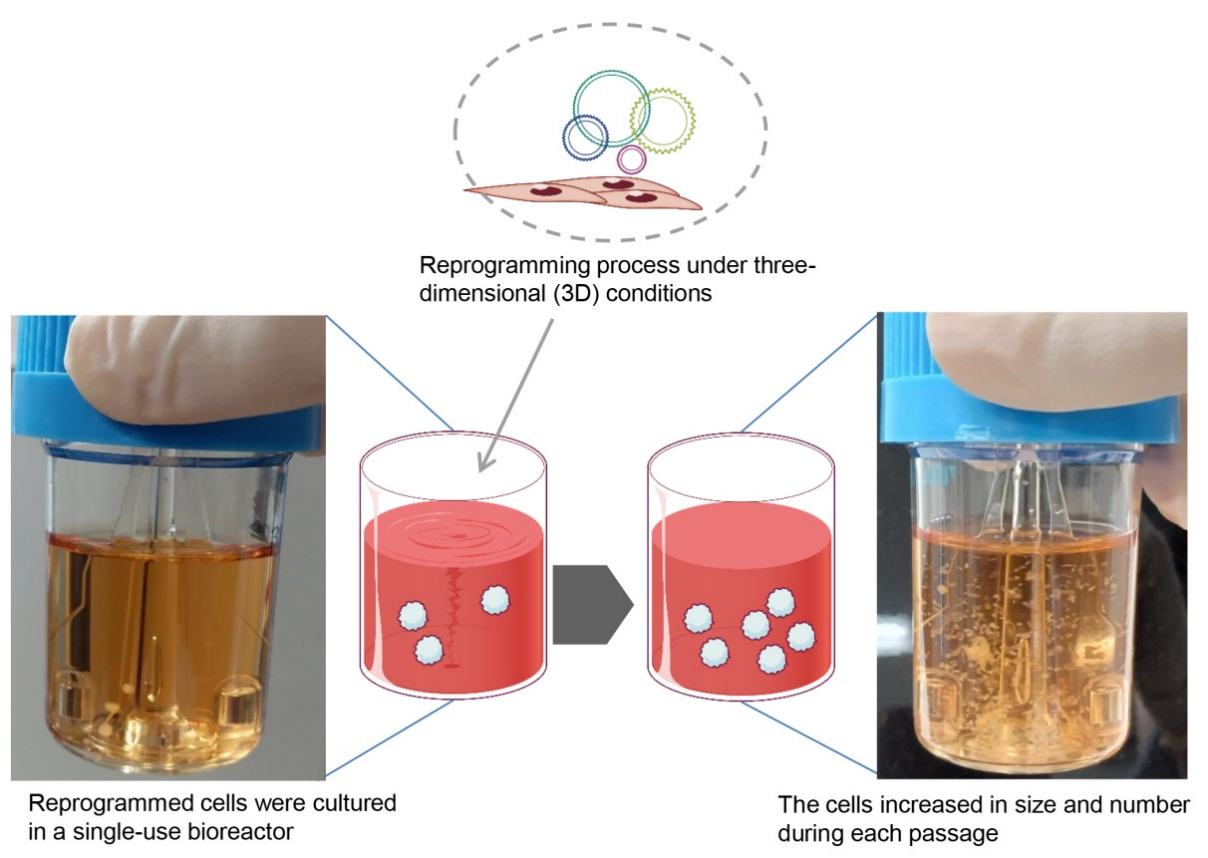


## Background

Induced pluripotent stem cells (iPSCs), derived from somatic cells, exhibit characteristics similar to those of embryonic stem cells. This makes them valuable tools in regenerative medicine and disease modeling [1]. However, the process of generating iPSCs is fraught with challenges, especially during the establishment phase, which requires specialized technical skills [2,3]. During the reprogramming phase, partially reprogrammed cells, re-differentiated cells, and non-reprogrammed cells all typically proliferate. However, a common issue is trying to attain spontaneous differentiation until cells achieve stable pluripotent states. It is therefore necessary to select and isolate iPSC colonies with optimal morphology during the initial passages. Such complexities contribute to the difficulties and inherent variability of iPSC cultures, which often depend on the technician's skills.

Traditionally, iPSCs are maintained under two-dimensional conditions using a specialized medium and feeder cells or an extracellular matrix reagent. This approach hampers scalability, which is crucial for iPSC downstream applications. Recently, the maintenance of iPSC lines under three-dimensional (3D) conditions has been shown to offer scalability. This has been achieved using agitation rotor machines or bioreactors [4,5]. However, the enzymatic dissociation step required for single-cell cultures under 3D conditions is both time-consuming and complex [4,6].

In particular, the generation of personalized iPSCs for use in regenerative medicine necessitates a simplified and streamlined workflow for iPSC studies. This workflow should minimize human handling during cell reprogramming, the maintenance of iPSCs, and their differentiation into specific cell types. Furthermore, it is also important that this simplified method can be scaled up to produce adequate cell mass.

To address these issues and reduce variability and workload for technicians, a more streamlined and convenient iPSC workflow is needed. We have developed the protocol presented here to enable iPSC generation under 3D culture conditions, thereby eliminating the need for a colony-picking step [7]. Moreover, our 3D culture system allows for the maintenance and cryopreservation of iPSCs without the need for enzymatic cell dissociation steps, resulting in a significantly simplified and easy-to-follow workflow. Given the compact size of the bioreactor used in this protocol (approximately 148 mm wide × 267 mm deep × 33 mm high with a maximum capacity of six samples at the same time), our protocol holds promise for the full automation of iPSC generation and maintenance in the future.

## Materials and reagents

**Biological materials**

1. Stealth RNA vector (SRV)^TM^ iPSC vector; SRV iPS-2 vector (encoding *OCT4, KLF4, SOX2*, and *C-MYC*; TOKIWA-Bio Inc., Ibaraki, Japan, catalog number: S1011694A) based on the Sendai virus
2. SRV^TM^ iPSC vector; SRV iPS-4 vector (carrying *OCT4, KLF4, SOX2, C-MYC, NANOG*, and *LIN28*; TOKIWA-Bio Inc., catalog number: S1011696A)
3. Adipose-derived mesenchymal stem cells (Lonza Bioscience, Walkersville, MD, USA, catalog number: PT5006)
4. Human peripheral blood mononuclear cells (PBMCs) (e.g., derived from healthy donors or patients)

**Reagents**
1. MesenPRO RS^TM^ medium (Thermo Fisher Scientific, catalog number: 12746012)
2. KBM 501 medium (Kohjin Bio, catalog number: 16025015)
3. Fetal bovine serum (FBS) (Thermo Fisher Scientific, catalog number: 10270-106)
4. TrypLE Select (Thermo Fisher Scientific, catalog number: 12563-011)
5. Dulbecco's phosphate-buffered saline (DPBS) (Thermo Fisher Scientific, catalog number: 14190144)
6. UltraPure^TM^ 0.5 M EDTA, pH 8.0 (Thermo Fisher Scientific, catalog number: 15575020)
7. StemScale^TM^ PSC suspension medium (StemScale) (Thermo Fisher Scientific, catalog number: A4965001)
8. DAPT, gamma-secretase inhibitor (Abcam, catalog number: ab120633)
9. EPZ004777 (iDOT1L) (Tocris, catalog number: 5567)
10. Y27632 (FUJIFILM Wako Pure Chemical Corporation, catalog number: 036-24023)
11. STEM-CELLBANKER^®^ GMP grade (ZENOAQ, catalog number: 11924)
12. RNeasy Mini kit (Qiagen, catalog number: 74904)
13. SuperScript IV VILO Master Mix (Thermo Fisher Scientific, catalog number: 11756050)
14. TaKaRa Ex Taq DNA Polymerase (Takara Bio Inc., catalog number: RR001B)
15. Primer pair for β-ACTIN and SRV (e.g., Sigma custom oligo; oligo sequence is below)
  - β-ACTIN (131 bp)
    Forward: 5-TCCCTGGAGAAGAGCTACG-3
    Reverse: 5-GTAGTTTCGTGGATGCCACA-3
  - SRV (500 bp)
    Forward: 5-ATATGGAGTACGAGAGGACC-3
    Reverse: 5-CCTCAGGTTGGAGAGAGTCA-3
16. Agarose gel (e.g., Nacalai Tesque Inc., catalog number: 01158-85)
17. Ethidium bromide (e.g., FUJIFILM Wako Pure Chemical Corporation, catalog number: 315-90051)

**Solutions**
1. Cell dissociation solution (see Recipes)
2. PBMC medium (see Recipes)
3. Reprogramming medium (see Recipes)

**Recipes**
1. **Cell dissociation solution**
  **Table:** 

Reagent	Final concentration	Amount
TrypLE Select	0.5×	25 mL
DPBS	0.5×	25 mL
EDTA	0.25 mM	25 µL
Total		50 mL
2. **PBMC medium**
  **Table:** 

Reagent	Final concentration	Amount
KBM 501 medium	90%	45 mL
FBS	10%	5 mL
Total		50 mL
3. **Reprogramming medium**
  **Table:** 

Reagent	Final concentration	Amount
StemScale	n/a	100 mL
DAPT	5 µM	
EPZ004777	3 µM	
Y27632	10 µM	
Total		100 mL
  n/a, not applicable

**Laboratory supplies**

1. Microtube 1.5 mL (e.g., Sumitomo Bakelite Co., Ltd., catalog number: MS-4265M)
2. 15 mL centrifuge tube (e.g., IWAKI, catalog number: 2324-015)
3. 30 mL disposable bioreactor (ABLE Biott, catalog number: ABBWVS03A-6)
4. 6-well dish (e.g., IWAKI, catalog number: 3810-006N)
5. 96-well U-bottom dish (e.g., Corning, catalog number: 7007)
6. PCR tube (e.g., Eppendorf, catalog number: 30124359)
7. Cryotube (e.g., Thermo Fisher Scientific, catalog number: 377267)

## Equipment

1. CO_2_ incubator (e.g., Thermo Fisher Scientific, catalog number: 3110, Forma^TM^ series II)
-80 °C freezer (e.g., Panasonic, catalog number: MDF-U33V-PJ)
3. Centrifuge (e.g., TOMY, catalog number: NIX521)
4. Bioreactor magnetic stir system base (Able Corp. & Biott Co., catalog number: ABBWBP03N0S-6)
5. Laser microscope (e.g., Keyence, catalog number: BZ-X810)
6. Thermal cycler (e.g., Thermo Fisher Scientific, catalog number: 4484073)
7. Cell counter (e.g., Beckman Coulter, Vi-CELL XR Cell Viability Analyzer System)

## Procedure

- **Transduction of reprogramming factors**
  1. Collection of somatic cells
    - When generating iPSCs from adipose-derived mesenchymal stem cells (AdSCs), collect the cells as a cell pellet after trypsinization with cell dissociation solution for 5 min at 37 °C and centrifugation at 200× *g* for 3 min.
    - Count the cells and resuspend them at a density of 1 × 10^7^/mL in 37 °C prewarmed MesenPRO RS^TM^ medium.
    - Transfer 200 µL of cell suspension (equivalent to 2 × 10^6^ cells) to a new 1.5 mL microtube.
*Note: If you attempt to reprogram somatic cells other than AdSCs, resuspend the cells in the appropriate culture medium for that specific cell type. For example, when we reprogram PBMCs, we resuspend the cells in PBMC medium.*
  2. Incubation of cells with Sendai virus vector
    - Thaw SRV iPS-2 vector, tap vigorously, briefly centrifuge at 1,000× *g* for 1–2 s at 25 °C, and then place on ice.
    - Add the SRV iPS-2 vector to the cell suspension at a multiplicity of induction (MOI) of 1.
    - Mix the solution by pipetting five times.
    - Incubate the cells with the SRV iPS-2 vector in a 1.5 mL microtube at 37 °C and 5% CO_2_ for 2 h.
    - Vigorously tap every 20 min.
*Notes:*
*i. Procedures involving viral vectors must be conducted in a Biosafety Level 2 (BSL2) laboratory or higher until SRV-removed iPSC lines are established. Appropriate safety guidelines and regulations for handling and disposal of biohazardous materials must be adhered to.*
*ii. Two types of SRV are commercially available. One carries four reprogramming factors: OCT4, SOX2, KLF4, and C-Myc. The other carries six reprogramming factors: the aforementioned four factors plus NANOG and LIN28A. The type of SRV to be used depends on the type of cells being reprogrammed. In our experience, fibroblasts can be reprogrammed with four-factor transduction, while PBMCs require six-factor transduction.*
*iii. The MOI is also a critical factor for successful reprogramming. In our experience, AdSCs can be reprogrammed with an MOI of 1, while PBMCs require an MOI of 3. We recommend starting the reprogramming experiment with an MOI of 1 or 3. If you are unable to reprogram other types of cells, you should optimize the MOI for efficient gene transduction.*
- **Reprogramming somatic cells under 3D conditions**
  1. Preparation of bioreactor for reprogramming
    - Add 20 mL of reprogramming medium to a 30 mL disposable bioreactor.
    - Pre-incubate at 37 °C, 5% CO_2_ on a bioreactor magnetic stir system base at 70 rpm placed within the incubator.
  2. Collection of reprogramming factor–transduced cells
    - After the 2 h incubation of AdSCs with the SRV iPS-2 vector, add 800 µL of MesenPRO RS^TM^ medium to the 1.5 mL microtube containing the cell–vector mixture. This brings the total volume of the cell suspension to 1 mL.
    - Immediately centrifuge the mixture at 300× *g* for 3 min and carefully aspirate the supernatant.
    - Gently resuspend the cell pellet in 1 mL of pre-warmed reprogramming medium.
    - Transfer the entire cell suspension to the bioreactor pre-placed inside the incubator. Incubate at 37 °C with 5% CO_2_, rotating at 70 rpm.
  3. Medium change
    - After 48 h, add 10 mL of reprogramming medium.
    - Another 48 h, collect 15 mL of cell suspension, centrifuge at 200× *g* for 3 min at 25 °C, and remove the supernatant.
    - Resuspend the cell pellet in 15 mL of fresh reprogramming medium.
    - Add the resuspended cells to the bioreactor.
    - Change half of the medium every 48 h until iPSC spheres emerge and grow.
    - Expect the appearance of some cell aggregates approximately 1-month post-seeding.
    - Continue changing the medium until the cells reach confluence. In this protocol, the iPSC aggregates increase in both size and number without requiring any single-cell dissociation steps.
    - Expect the cells to reach confluence approximately 2–3 weeks after the primary cell aggregates emerge.
- **Establishment of iPSC lines under 3D conditions**
  1. Preparation of bioreactor for maintenance
    - Add 30 mL of StemScale (without DAPT, iDOT1L, or Y27632) to a new 30 mL bioreactor.
    - Pre-incubate at 37 °C, 5% CO_2_ on a bioreactor magnetic stir system base at 70 rpm.
  2. Selection of SRV vector-negative iPSC spheres (Figure 1)
    - Once the primary iPSC spheres reach confluence, transfer from 5 to 10 mL of spheres into a 6-well dish.
    - Under a microscope and using a P1000 pipette, pick up each small sphere (typically ranging in size from 200 to 300 µm) and place it into a well of a 96-well dish. It is important to ensure that no more than one sphere is placed into each well.
    - Detect the green fluorescent protein (GFP) signal using a laser microscope and mark wells containing GFP-negative spheres, as the SRV iPS-2 vector encodes the *EGFP* gene in its backbone.
    - Expect to obtain 5–10 GFP-negative spheres from one 96-well dish. Transfer only GFP-negative spheres to a new 30 mL bioreactor containing StemScale.
    - Change the medium every 48 h until the iPSC spheres increase in size and number.
    **Figure 1.**
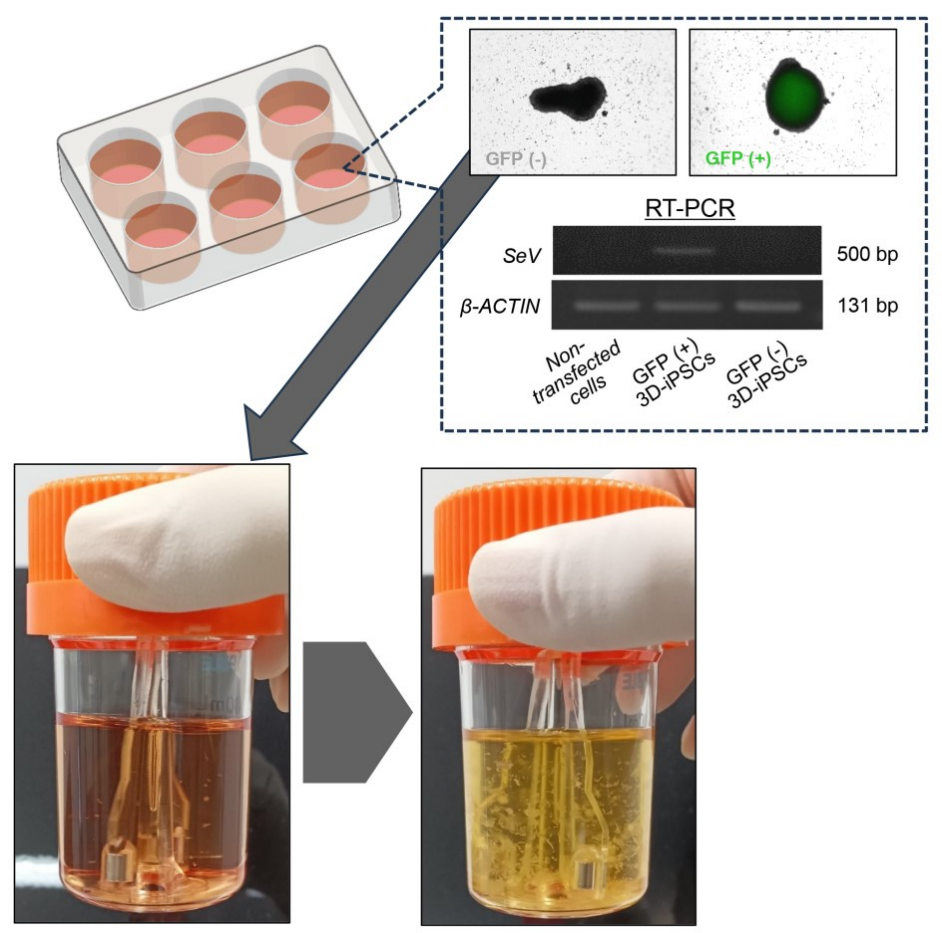
Selection and expansion of reprogramming virus vector-free induced pluripotent stem cells (iPSCs) in suspension culture. Cells were observed by fluorescence microscopy for green fluorescent protein (GFP) expression (indicating Sendai virus vector remnant cells). Cells without residual GFP expression were selectively transferred to the next bioreactor. The cells increased in size and number during each passage. GFP-negative spheres indicate the absence of Sendai virus (SeV), as shown by quantitative reverse transcription polymerase chain reaction (RT-PCR) image (right panel). *β-ACTIN* was the housekeeping gene.
  3. Verification of exogeneous reprogramming vector removal
    - Extract total RNA from GFP-positive and GFP-negative iPSC spheres and parental somatic cells.
    - Perform a reverse transcription (RT) reaction using RT enzyme (e.g., Super Script IV Vilo Master Mix).
    - Perform a polymerase chain reaction (PCR) for a housekeeping gene such as *β-ACTIN* and the SRV vector. PCR amplification is performed with the appropriate primer pairs and TaKaRa Ex Taq DNA polymerase under the following conditions: initial denaturation at 98 °C for 10 s, followed by 35 cycles of denaturation at 98 °C for 10 s, annealing at 60 °C for 30 s, and extension at 72 °C for 45 s. PCR products are resolved on a 2% agarose gel containing ethidium bromide.
- **Maintenance of iPSCs**
  1. Preparation of bioreactor for maintenance (Figure 2)
    - Add 30 mL of StemScale (without DAPT, iDOT1L, or Y27632) to a new 30 mL bioreactor.
    - Pre-incubate at 37 °C, 5% CO_2_ on a bioreactor magnetic stir system base at 70 rpm.
    **Figure 2.**
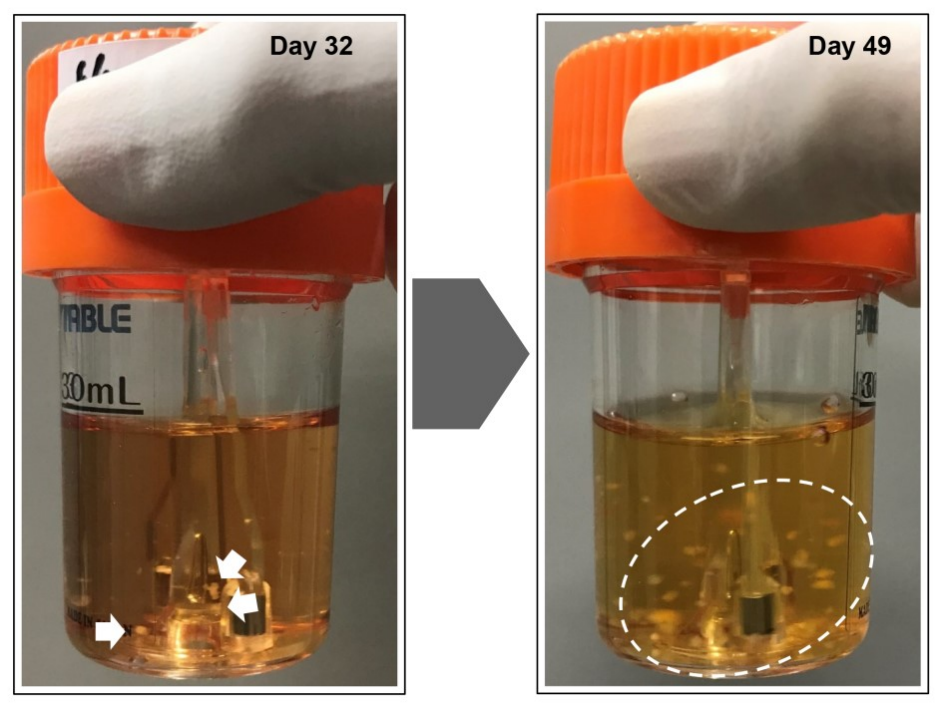
Reprogramming of somatic cells into induced pluripotent stem cells (iPSCs) in a suspension culture. The reprogrammed cells emerged as small clumps of cells (white arrows) in a spinner flask culture. The spheroids increased in size, cleaved into smaller clumps (white dashed circle), and self-dissociated into smaller pieces over time. The formation of reprogrammed spheroids was observed approximately 30 days after initiating the reprogramming process.
  2. Passage of iPSCs
    - Transfer a few iPSC spheres (usually 10 spheres are enough) to a new 30 mL bioreactor using a P1000 pipette or 5 mL autopipettor. While our preferred size is approximately 500 µm in diameter, spheres of any size can be transferred.
    - Incubate at 37 °C and 5% CO_2_ at 70 rpm, and perform a half-medium change every 48 h until iPSCs reach confluence.
- **Cryopreservation of 3D-cultured iPSCs**
  1. Cell collection
    - After cell growth, transfer 5 mL of spheroid suspension to a 15 mL centrifuge tube.
    - Centrifuge the 15 mL tube at 300× *g* for 3 min or simply let the tube stand for 3 min.
    - Remove the supernatant.
  2. Cryopreservation
    - Suspend the iPSC spheres in 5 mL of STEM-CELLBANKER medium, pre-chilled to 4 °C.
    - Immediately transfer 1 mL of the cell suspension to each cryopreservation tube.
    - Cryopreserve the cells at -80 °C for short-term storage. For long-term storage, preserve at -276 °C.

## Data analysis

As this protocol describes the generation and maintenance of iPSCs under 3D conditions, data analysis was not necessary.

## Validation of protocol

This protocol has been validated for the generation of iPSCs from PBMCs. PBMCs were isolated from donors, and 2 × 10^6^ cells were incubated with the SRV iPS vector. For the reprogramming of PBMCs, we employed the SRV iPS-4 vector, which encodes six reprogramming factors. By cultivating the six-factor transduced PBMCs in the reprogramming medium, we observed the emergence of primary iPSC spheres approximately 25 days post-seeding. After cultivating the primary spheres in the same reactor used for reprogramming, the primary iPSC spheres expanded in both size and number without the need for a single-cell dissociation step. Following the selection of GFP-negative spheres and confirmation of transgene removal, we were able to successfully maintain these PBMC-derived iPSCs using the outlined technique. PBMC-derived iPSCs exhibited undifferentiated states and demonstrated the capability to differentiate into all three germ layers, both in vitro and in vivo.

This protocol or parts of it has been used and validated in the following research article(s):

- Tsukamoto et al. [7]. A passage-free, simplified, and scalable novel method for iPSC generation in three-dimensional culture. *Regenerative Therapy* (Figure 4).

## General notes and troubleshooting

**General notes**

1. A limitation of this protocol lies in the manual selection of GFP-negative spheres under the microscope. This step is labor-intensive and time-consuming compared with other steps in this protocol.
2. The undifferentiated state of the iPSCs produced and maintained using this protocol is evaluated through standard quantitative PCR and immunostaining procedures. After paraffin-embedding and sectioning, immunostaining can be performed.
3. The differentiation potential of iPSCs can be assessed in vitro by embryoid body formation and in vivo by teratoma formation.

**Troubleshooting**

**Table:** 

Issue	Suggested solution
No iPSC spheres observed	Ensure that the AdSCs do not undergo senescence. Cellular senescence and low growth activity can hinder efficient cell reprogramming. Verify that the SRV vector was stored at -80 °C or below. We recommend making aliquots to avoid multiple freeze-thaw cycles. Check that the reprogramming medium is fresh. Use fresh reprogramming medium supplemented with DAPT and iDOT1L. We recommended making aliquots of the StemScale medium and using it within two weeks.
No transgene silencing (no GFP-negative spheres)	There are four types of commercially available SRV vectors. The SRV used in this study is either SRV iPS-2 or iPS-4, which are automatically removed from reprogrammed cells by endogenous microRNA-302. If you are using SRV iPS-1 or iPS-3, a small interfering (si)RNA procedure should be performed to remove the SRV from the iPSCs.
